# Comparison of Various Methods to Determine Added Sugars Intake to Assess the Association of Added Sugars Intake and Micronutrient Adequacy

**DOI:** 10.3390/nu12092816

**Published:** 2020-09-14

**Authors:** Victor L. Fulgoni, P. Courtney Gaine, Maria O. Scott

**Affiliations:** 1 Nutrition Impact, LLC, Battle Creek, MI 49014, USA; 2 The Sugar Association, Inc., Washington, DC 20005, USA; gaine@sugar.org (P.C.G.); mscott@sugar.org (M.O.S.)

**Keywords:** added sugars, intake methods, micronutrient adequacy, the NHANES

## Abstract

Different methods for determining the effect of added sugars intake among children and adults on meeting recommended nutrient intakes were compared using 24 h dietary recall data from the National Health and Nutrition Examination Survey (NHANES) 2011–2014. Four methods were used to determine deciles of added sugars intake (as the percentage of total calories): 1 day intake, 2 day average intake, and individual usual intake (UI) determined with the National Cancer Institute (NCI) and the multivariate Markov Chain Monte Carlo methods. Percentages of the population below the Estimated Average Requirement (EAR) for calcium and vitamin D/above the Adequate Intake (AI) for potassium and dietary fiber for each decile of added sugars intake were assessed with the NCI method. Using regression analyses, added sugars intake deciles (by any method) in children were inversely associated (*p* < 0.001) with percentages below the EAR/above the AI of vitamin D, calcium, potassium, and fiber. In adults, added sugars intake deciles were inversely associated with meeting recommendations for vitamin D, potassium, and fiber. There were no significant between-method differences for regression coefficients for any nutrients investigated. Overall, these methods showed a similar association of added sugars intake with nutrient inadequacy/adequacy; therefore, method preference may depend more on practical reasons.

## 1. Introduction

Nutrition and health surveys play an important role in assessing and monitoring dietary patterns and trends, assist in the analysis of dietary factors related to disease risk, and provide background data for informing nutrition policy. The National Health and Nutrition Examination Survey (NHANES) is designed to monitor the health and nutritional status of the U.S. population. NHANES data are used to determine the prevalence of diseases and their risk factors, assess nutritional status and its association with health promotion and disease prevention, and assist with formulation of national standards and public health policy [1]. The Centers for Disease Control and Prevention National Center for Health Statistics (NCHS) conducts the NHANES every year using a stratified, multistage probability design to obtain a nationally representative sample. Demographic and basic health information data are collected via an in-home interview, and participants complete a comprehensive diet and health examination at a Mobile Examination Center. Dietary intake information is collected via the Automated Multiple-Pass Method [2] using two 24 h dietary recalls, the first conducted in person in the Mobile Examination Center interview (day 1 recall) and the second via telephone interview 3–10 days later (day 2 recall). 

In addition to 24 h dietary recall (single or multiple days), food frequency questionnaires (FFQs) and dietary records are other commonly used methods to assess dietary intake in nutritional surveys [3,4,5,6]. Long-term or habitual intakes, typically referred to as usual intake (UI), are estimated from short-term measurements using various modeling techniques that take into account within-person variation. Various statistical methodologies using a similar general approach have been developed for estimating UI distributions [7,8,9,10,11,12,13]. Nevertheless, there are differences between the methods used and their underlying assumptions. To date, the different methods of UI estimation, especially the newly developed methods, have only been compared in a few reports and have used only simulation studies [14,15,16]. Therefore, the objective of the present study was to compare dietary intake estimation by four approaches: 1 day and 2 day average 24 h dietary recall and UI determined with the National Cancer Institute (NCI) and the Markov Chain Monte Carlo (MCMC) methods. The MCMC method allows for simulation of changes in more than one variable (e.g., nutrient intake and calories). Intake of added sugars and its effect on nutrient adequacy/inadequacy were used as an example, as authoritative public health organizations from around the world recommend limiting intake of added sugars [17,18] due to the potential association of added sugars intake with decreased diet quality and increased chronic disease risk [19,20,21,22,23]. 

## 2. Materials and Methods

### 2.1. Data Source and Participants

Data from the NHANES 2011–2014 from 5876 children aged 2–18 years and 9953 adults aged ≥19 years, excluding pregnant and lactating females (*n* = 184) and those with unreliable dietary records as determined by NCHS staff (*n* = 2640), were used for the current analysis [1]. NCHS/USDA codes recalls as reliable if the first 4 steps of the 5-step Automated Multiple Pass Method of dietary recall collection were completed and all foods/beverages reported for each eating occasion have been identified.

Written informed consent was obtained from all participants directly or through parental/guardian proxies, and the NHANES protocol was approved by the NCHS Ethics Review Board. The NHANES has stringent protocols and procedures to ensure confidentiality and protect identification of individual participants using federal laws; therefore, additional institutional review board approval for these secondary analyses was not required. Detailed descriptions of the NHANES sample design, interview procedures, and survey response rates are available online (https://www.cdc.gov/nchs/nhanes/about_nhanes.htm). 

### 2.2. Estimation of Added Sugars Intake

Intake of added sugars was determined as the percentage of total calories (% kcal) using the added sugars values in the U.S. Department of Agriculture Food Patterns Equivalent Database (FPED) for each NHANES release [24]. The FPED defines added sugars as “sugars that are added to foods as an ingredient during preparation, processing, or at the table” and does not include naturally occurring sugars such as lactose present in milk and fructose present in whole or cut fruit or 100% fruit juice. However, fruit juice concentrates used as ingredients are assigned to added sugars. In this study, deciles of added sugars intake (% kcal) were calculated using the following methods: (1) 1 day 24 h dietary recall data, (2) 2 day average of 24 h dietary recall data, (3) individual UI as determined by the NCI method version 2.1 (UI/NCI) [9], and (4) UI as determined by the multivariate MCMC method—an alternative to the NCI method (UI/MCMC) [13] that allows for, in this analysis, simultaneous simulation of both added sugars calories and total calories. 

### 2.3. Intake of Micronutrients 

Intakes of underconsumed “nutrients of public health concern” (calcium, potassium, dietary fiber, and vitamin D) [17] were estimated as UI using only the NCI method for each decile of added sugars intake for each of the four methods to define added sugars intake. Adequacy of population micronutrient intakes for each intake decile of added sugars was estimated as the percentage of the population below the Estimated Average Requirement (EAR) for calcium and vitamin D/above the Adequate Intake (AI) for potassium and dietary fiber. 

### 2.4. Statistical Analyses 

SAS 9.4 software (SAS Institute, Cary, NC) was used for all statistical analyses. The data were adjusted for the complex sampling design of the NHANES, using appropriate survey weights, sampling parameter strata, and primary sampling units. Separate analyses were performed for children aged 2–18 years and for adults aged ≥19 years. The one-part model was used to determine the UI of micronutrients using the NCI method. Two days of intake by 24 h dietary recalls and 1 day sampling weights were used to obtain percentiles of intake and necessary variance estimates; models included day of the week of the 24 h recall and sequence of dietary recall as covariates [9]. Briefly, the observed intake data from 24 h recalls were Box–Cox transformed and fitted into a linear mixed-effects model to estimate mean and within- and between-individual variances. The distribution of UI based on the estimated variance was empirically estimated and data were then back transformed. For the UI/MCMC method [13], the two 24 h recall intake data sets were Box–Cox transformed and then added sugars calories and total energy intake were simultaneously modeled; 500 replications for each individual were performed, and the mean values of replications per individual were used to assign deciles of intake with this method. Thus, a multivariable version of the MCMC method was used to fit a multivariate measurement error model for added sugars and calories, and the UI distribution of the added sugars percentage of total calories was estimated. The SAS macro MULTIVAR_MCMC was used. The covariates Dietary Reference Intake age groups, gender, weekend/non-weekend intake, and day sequence were used in the estimation. The SAS macros and example programs are available on the NCI website (http://appliedresearch.cancer.gov/diet/usualintakes/macros.html). A user guide for the NCI SAS macros is also available online (https://epi.grants.cancer.gov/diet/usualintakes/users_guide_multivariate_mcmc_distrib_brr_macros_v1.0.pdf).

Regression analyses were conducted to examine potential relationships between decile of added sugars intake and micronutrient adequacy using dummy-coded deciles of intake (deciles 1–10) as the independent variable for all four methods of measuring added sugars intake. Regression coefficients (measuring change in nutrient adequacy for each change in decile of added sugars intake) were generated to examine the direction and magnitude of any relationships. Given our large sample size and in lieu of adjustment for multiple comparisons, a conservative *p* value of ≤ 0.001 was used to assess whether the regression coefficient was different from zero. Non-overlapping confidence intervals were used to assess whether the different methods of measuring intake provide different regression coefficients.

## 3. Results

### 3.1. Added Sugars Deciles 

For children aged 2–18 years, the deciles of added sugars intake (% kcal) ranged from ≤5.2 to >25.8 using 1 day intake, ≤6.4 to >22.6 using 2 day average intake, ≤10.8 to >18.2 using the UI/NCI method, and ≤12.1 to >15.0 using the UI/MCMC method (Table 1). Median intake of added sugars for children was 13.5% kcal based on 1 day intake, 13.0% kcal based on 2 day average intake, 14.2% kcal based on the UI/NCI method, and 13.8% kcal based on the UI/MCMC method. 

For adults aged ≥19 years, the deciles of added sugars intake (% kcal) ranged from ≤2.6 to >25.3 using 1 day intake, ≤3.8 to >22.9 using 2 day average intake, ≤6.8 to >19.4 using the UI/NCI method, and ≤11.1 to >13.7 using the UI/MCMC method (Table 1). Median intake of added sugars for adults was 11.1% kcal based on 1 day intake, 10.8% kcal based on 2 day average intake, 11.9% kcal based on the UI/NCI method, and 12.5% kcal based on the UI/MCMC method.

### 3.2. Calcium Intake Inadequacy by Deciles of Added Sugars 

For children aged 2–18 years, there were significant associations (*p* ≤ 0.001) between decile of added sugars intake using 2 day average intake, the UI/NCI method, or the UI/MCMC method and calcium intake inadequacy (the percentage of the population below the EAR). For each 1-decile increase in added sugars intake, calcium intake inadequacy increased by 4.19% for 2 day average intake, 4.89% for the UI/NCI method, and 7.85% for the UI/MCMC method. There was no significant association (*p* > 0.001) between decile of added sugars intake using 1 day intake and calcium intake inadequacy (Table 2). 

For adults aged ≥19 years, there were no significant associations (*p* > 0.001) between decile of added sugars intake using 1 day intake, 2 day average intake, the UI/NCI method, or the UI/MCMC method and calcium intake inadequacy (Table 2).

### 3.3. Potassium Intake by Deciles of Added Sugars

For children aged 2–18 years, there were significant inverse associations (*p* ≤ 0.001) between decile of added sugars intake using 1 day intake, 2 day average intake, or the UI/NCI method and potassium intake (the percentage of the population above the AI). For each 1-decile increase in added sugars intake, potassium intake as the percentage above the AI decreased by 0.16% for 1 day intake, 0.15% for 2 day average intake, and 0.13% for the UI/NCI method. There was no significant association (*p* > 0.001) between decile of added sugars intake by the UI/MCMC method and potassium intake as the percentage above the AI (Table 3). 

For adults aged ≥19 years, there were significant inverse associations (*p* ≤ 0.001) between decile of added sugars intake using 2 day average intake or the UI/NCI method and potassium intake (percentage above the AI). For each 1-decile increase in added sugars intake, potassium intake as the percentage above the AI decreased by 0.47% for 2 day average intake and by 0.42% for the UI/NCI method. There were no significant associations (*p* > 0.001) between decile of added sugars intake using 1 day intake or the UI/MCMC method and potassium intake as the percentage above the AI (Table 3).

### 3.4. Dietary Fiber Intake by Deciles of Added Sugars

For children aged 2–18 years, there were significant inverse associations (*p* ≤ 0.001) between decile of added sugars intake using 1 day intake, 2 day average intake, or the UI/NCI method and dietary fiber intake (the percentage of the population above the AI). For each 1-decile increase in added sugars intake, dietary fiber intake as the percentage above the AI decreased by 0.21% for 1 day or for 2 day average intake and 0.20% for the UI/NCI method. There was no significant association (*p* > 0.001) between decile of added sugars intake by the UI/MCMC method and dietary fiber intake as the percentage above the AI (Table 4).

For adults aged ≥19 years, there were significant inverse associations (*p* ≤ 0.001) between decile of added sugars intake using 1 day intake, 2 day average intake, the UI/NCI method, or the UI/MCMC method and dietary fiber intake (the percentage of the population above the AI). For each 1-decile increase in added sugars intake, dietary fiber intake as the percentage above the AI decreased by 1.43% for 1 day intake, 1.66% for 2 day average intake, 1.54% for the UI/NCI method, and 1.39% for the UI/MCMC method (Table 4).

### 3.5. Vitamin D Intake Inadequacy by Deciles of Added Sugars

For children aged 2–18 years, there were significant associations (*p* ≤ 0.001) between decile of added sugars intake using 1 day intake, 2 day average intake, or the UI/NCI method and vitamin D intake inadequacy (the percentage of the population below the EAR). For each 1-decile increase in added sugars intake, vitamin D intake inadequacy increased by 1.22% for 1 day intake, 1.29% for 2 day average intake, and 1.32% for the UI/NCI method. There was no significant association (*p* > 0.001) between decile of added sugars intake for the UI/MCMC method and vitamin D intake inadequacy (Table 5).

For adults aged ≥19 years, there were no significant associations between decile of added sugars intake using 1 day intake, 2 day average intake, and the UI/NCI method and vitamin D intake inadequacy. However, there was a significant association (*p* ≤ 0.001) between decile of added sugars intake using the UI/MCMC method and vitamin D intake inadequacy (the percentage of the population below the EAR). For each 1-decile increase in added sugars intake, vitamin D intake inadequacy increased by 0.37% (Table 5).

### 3.6. Comparison of Methods

Table 6 presents the regression coefficients of the percentage of the population below the EAR/above the AI of nutrients and of added sugars deciles by different methods. In general, there were no significant differences (non-overlapping 97.5% confidence interval) between the four methods used to develop deciles of added sugars intake for the regression coefficients for any of the four nutrients investigated for children or adults. However, in adults, only the regression coefficient for the percentage of the population below the EAR for calcium as added sugars intake increased for the MCMC methods was significantly larger than that for the other methods.

## 4. Discussion

Micronutrient dilution of the diet at higher added sugars intakes has been cited as a concern by public health organizations recommending limits on added sugars intake [17,18]. However, the evidence is not consistent and may be limited by methodological constraints [25,26,27,28,29,30,31]. In this report, we analyzed the association of added sugars intake with the percentage of the population below the EAR of calcium and vitamin D intake/above the AI of potassium and dietary fiber intake. We chose these nutrients because their current intake is very low and they have been characterized as “nutrients of public health concern” [17]. The sufficiency or insufficiency of micronutrient intakes along the continuum of added sugars intake was examined using deciles of added sugars instead of arbitrarily selecting set points. We used four different methods of estimating deciles of added sugars intakes (1 day 24 h dietary recall data, 2 day average 24 h dietary recall data, the UI/NCI method, and the UI/MCMC method) and compared them for the association of added sugars deciles with micronutrient intake as the percentage of the population below the EAR/above the AI. It is interesting to note that all methods generally yielded a significant direct association between deciles of added sugars and inadequacy of calcium and vitamin D intakes, and there was an inverse association between deciles of added sugars and the percentage above the AI of potassium and dietary fiber intakes for children or adults. However, except for calcium in adults, there were no significant differences between the four different methods. With all four methods, those with the lowest added sugars intake generally still had a large percentage of the population below the EAR for calcium and vitamin D, as reported previously [30,31]. These findings indicate that obtaining the recommended intake of these nutrients is not solely related to added sugars intake but rather to consumption of specific foods high in these nutrients.

The results show that both the range and the median of added sugars deciles in children and adults were dependent on the method used to estimate added sugars deciles. As expected, the largest range in deciles of the added sugars (as % kcal) in both children and adults was highest for the 1 day intakes (~20 percentage units), reduced somewhat for the average of 2 day intakes (~15 percentage units), and decreased further with UI methods (NCI: ~8 percentage units; MCMC: ~3 percentage units). Additionally, the difference in the first decile of added sugars intake among the methods in children was ≤5.2, ≤6.4, ≤10.8, and ≤ 12.1% of calories for the 1 day, 2 day, UI/NCI, and UI/MCMC methods, respectively; comparable data for the highest decile were >25.8, >22.6, >18.2, and >15.0% calories. A similar pattern was also seen in adults (first decile: ≤2.6, ≤3.8, ≤6.8, and ≤ 11.1% of calories for the 1 day, 2 day, UI/NCI, and UI/MCMC methods, respectively; comparable data for the highest decile were >25.3, >22.9, >19.4, and >13.7% calories). There were significant associations between the percentage of the population below the EAR/above the AI of nutrient intakes and added sugars deciles for some nutrients and for some methods. However, there were very few significant between-method differences for the associations of added sugars intake and the percentage of the population below the EAR/above the AI. The only difference in regression coefficients across methods was seen in the relationship of calcium adequacy in adults for the UI/MCMC method (e.g., the percentage of the population below the EAR increased 3.4 percentage units per decile of added sugars intake, with other methods indicating that the regression coefficient was 1% or less). While this difference may be due to chance, it is also likely that because calcium intake was based, at least somewhat, on total calories, the UI/MCMC method may have greater sensitivity to assess associations when nutrient intake is strongly correlated with calorie intake.

Self-reported 24 h dietary recall assesses an individual’s consumption in the past 24 h and can be used to characterize the average consumption of a group of individuals for that day. In any self-reported dietary assessment instrument, there is an inherent measurement error that leads to biased estimates; therefore, for reliable interpretation, validity of such self-reported instruments is needed [32]. Additionally, since there are day-to-day variations in an individual’s diet due to various biological and environmental influences, a single 24 h recall is not representative of an individual’s long-term intake [33,34]. Intraindividual variability can be eliminated to some extent by using repeated 24 h dietary recalls, especially if the data are collected randomly on non-consecutive days. However, there may be methodological inconsistences in collecting data on multiple days (e.g., data collection via an in-person interview on day 1 and via telephone interview on day 2). For dietary assessments in national dietary surveys, 24 h dietary recall methods are most commonly used and are preferred over dietary record/history and FFQ methods [4,35]. However, long-term habitual dietary intake or “UI” of the population is of interest to scientists and policy makers because dietary recommendations are intended to be achieved over time. Habitual intakes are also useful in identifying population groups who are at risk of having an inadequate/insufficient dietary intake or excessive consumption. The distribution of 24 h (single or mean of multiple) dietary intakes of a representative sample is used to estimate UI and the proportion of the population with intakes above or below particular dietary standards [36]. Statistical modeling of the short-term measurement data is needed to address intra- and interindividual variations, and various statistical methodologies have been proposed for estimating UI distributions [7,8,9,10,11,12,13]. The UI/NCI method can be used to estimate the distribution of UI of both occasionally and regularly consumed nutrients and foods. The UI/NCI method provides good estimates of the UI distributions; however, the estimates are less accurate for small sample sizes [14,15]. The UI/MCMC method is an alternative to the UI/NCI method incorporating the estimation of energy intake into the model [13]. This method is more complex and uses the MCMC simulation approach instead of maximum likelihood. Although the UI/MCMC method is fairly reliable, it can be very difficult to assess accuracy and obtain convergence. It is interesting to note that, in general, all four methods of short- and long-term intake estimation provided a similar association of deciles of added sugars intake with the percentage of the population below the EAR/above the AI. While there were large differences in the range of intakes of added sugars as a percentage of calories across the methods, the relationship of added sugars to adequacy of the nutrients evaluated was relatively similar across methods. The median intakes measured with each method were also relatively similar (11.1, 10.8, 11.9, and 12.5% kcal for 1 day intake, 2 day average intake, UI/NCI method, and UI/MCMC method, respectively) but given intra- and interindividual variation mentioned above, to estimate the percentage of the population above added sugars recommendations, one of the UI methods is recommended.

There are some limitations to our research that should be considered when drawing conclusions: (1) all of our intake measures are subject to self-reporting issues associated with 24 hr recalls; (2) we used a conservative *p* value (i.e., <0.001) given our large sample size and propensity to thus detect very small numerical differences, and this may have also lead to concluding non-significance where another *p* value may have shown a significant association; (3) we only examined four nutrients and two of the four nutrients we examined have either a very high (<90%) percentage of the population below the EAR or a very low (<5%) percentage of the population above the AI; and (4) as our focus was on comparing methods of measuring added sugars intake, no attempt was made to adjust for total caloric intake or diet quality which are known to impact nutrient adequacy.

While we would expect analyses using deciles of intake to help mediate issues such as skewness/non-normality and a non-linear relationship of a nutrient along the range of intakes of added sugars, it is possible that when a large number of subjects are sorted into numerous groups (e.g., deciles of intake as used in this study), some of the weaknesses of shorter-term measures of intake are minimized such that similar relationships across intake levels are obtained. That said, as each of the methods offer different features and complexities, preference for one method over the others may depend more on practical reasons such as availability of data and computing capacity. However, more examples need to be investigated to confirm these findings.

## Figures and Tables

**Table 1:** Deciles of added sugars intake (% kcal) of the U.S. population by various methods, the NHANES 2011–2014. ^1^

Decile ^2^	1 Day Intake	2 Day Average Intake	UI/NCI	UI/MCMC
Children (2–18 years)				
1	≤5.2	≤6.4	≤10.8	≤12.1
2	>5.2 to ≤7.5	>6.4 to ≤8.5	>10.8 to ≤12.0	>12.1 to ≤12.7
3	>7.5 to ≤9.5	>8.5 to ≤10.0	>12.0 to ≤12.9	>12.7 to ≤13.2
4	>9.5 to ≤11.5	>10.0 to ≤11.5	>12.9 to ≤13.6	>13.2 to ≤13.5
5	>11.5 to ≤13.5	>11.5 to ≤13.0	>13.6 to ≤14.2	>13.5 to ≤13.8
6	>13.5 to ≤15.6	>13.0 to ≤14.8	>14.2 to ≤15.0	>13.8 to ≤14.0
7	>15.6 to ≤17.8	>14.8 to ≤16.8	>15.0 to ≤15.8	>14.0 to ≤14.2
8	>17.8 to ≤20.8	>16.8 to ≤19.0	>15.8 to ≤16.8	>14.2 to ≤14.5
9	>20.8 to ≤25.8	>19.0 to ≤22.6	>16.8 to ≤18.2	>14.5 to ≤15.0
10	>25.8	>22.6	>18.2	>15.0
Adults (≥19 years)				
1	≤2.6	≤3.8	≤6.8	≤11.1
2	>2.6 to ≤4.9	>3.8 to ≤5.8	>6.8 to ≤8.5	>11.1 to ≤11.3
3	>4.9 to ≤7.0	>5.8 to ≤7.5	>8.5 to ≤9.8	>11.3 to ≤11.5
4	>7.0 to ≤9.0	>7.5 to ≤9.2	>9.8 to ≤10.8	>11.5 to ≤12.0
5	>9.0 to ≤11.1	>9.2 to ≤10.8	>10.8 to ≤11.9	>12.0 to ≤12.5
6	>11.1 to ≤13.6	>10.8 to ≤12.8	>11.9 to ≤13.2	>12.5 to ≤12.7
7	>13.6 to ≤16.3	>12.8 to ≤15.1	>13.2 to ≤14.7	>12.7 to ≤13.0
8	>16.3 to ≤19.5	>15.1 to ≤18.1	>14.7 to ≤16.5	>13.0 to ≤13.3
9	>19.5 to ≤25.3	>18.1 to ≤22.9	>16.5 to ≤19.4	>13.3 to ≤13.7
10	>25.3	>22.9	>19.4	>13.7

^1^ UI/MCMC, usual intake by the Markov Chain Monte Carlo method; UI/NCI, usual intake by the National Cancer Institute method; NHANES, National Health and Nutrition Examination Survey. ^2^ Deciles were calculated as 100 × ratio intake of added sugars (kcal) to intake of total calories (kcal) estimated by the same method. Data source for added sugars: USDA Food Patterns Equivalent Database 2011–2012 and 2011–2014.

**Table 2:** Percentage of the population below the Estimated Average Requirement of calcium by deciles of added sugars intake by various methods, the NHANES 2011–2014. ^1^

Decile	1 Day Intake	2 Day Average Intake	UI/NCI	UI/MCMC
Children (2–18 years)				
1	40.8 ± 3.7	32.7 ± 3.9	27.2 ± 3.2	4.41 ± 0.70
2	28.9 ± 2.8	28.3 ± 2.1	25.2 ± 2.2	32.6 ± 1.9
3	26.5 ± 2.5	33.3 ± 2.7	27.7 ± 3.3	50.0 ± 2.9
4	30.0 ± 2.5	30.1 ± 3.0	35.2 ± 2.5	29.5 ± 3.1
5	33.3 ± 2.4	34.6 ± 3.4	32.9 ± 2.5	48.2 ± 2.2
6	41.4 ± 3.7	43.4 ± 3.7	42.7 ± 3.6	45.8 ± 3.3
7	45.8 ± 4.5	47.3 ± 4.1	47.4 ± 3.6	45.4 ± 4.0
8	47.6 ± 2.7	42.1 ± 4.4	49.2 ± 4.3	53.5 ± 4.0
9	59.3 ± 3.6	59.5 ± 3.8	60.4 ± 3.7	53.6 ± 5.1
10	73.7 ± 4.1	75.5 ± 4.3	75.7 ± 4.0	63.1 ± 5.9
β ^2^	4.04 ± 0.94	4.19 ± 0.75	4.89 ± 0.62	7.85 ± 1.48
*p* ^3^	0.0027	0.0005	0.0001	0.0007
Adults (≥19 years)				
1	52.7 ± 2.4	52.4 ± 2.5	54.9 ± 2.0	56.1 ± 2.7
2	42.9 ± 2.3	44.5 ± 2.6	45.6 ± 1.8	55.1 ± 1.6
3	34.9 ± 2.8	34.3 ± 2.6	36.3 ± 2.6	51.2 ± 2.0
4	37.2 ± 2.2	36.8 ± 2.4	36.7 ± 2.6	58.7 ± 2.2
5	36.2 ± 2.1	37.5 ± 2.4	35.7 ± 3.0	40.8 ± 2.0
6	34.7 ± 2.1	33.9 ± 2.2	34.3 ± 2.1	32.0 ± 2.3
7	39.3 ± 2.7	40.4 ± 1.9	38.8 ± 1.9	29.9 ± 2.6
8	39.2 ± 2.6	40.4 ± 2.2	37.7 ± 1.9	30.9 ± 2.2
9	47.4 ± 1.9	45.1 ± 2.2	42.7 ± 2.4	31.4 ± 1.7
10	60.4 ± 2.0	62.0 ± 2.6	61.5 ± 2.1	39.0 ± 2.9
β ^2^	1.10 ± 0.96	0.62 ± 0.97	0.04 ± 1.03	−3.40 ± 0.70
*p* ^3^	0.2844	0.5444	0.9719	0.0012

^1^ Values are presented as the mean ± SD unless otherwise indicated. UI/MCMC, usual intake by the Markov Chain Monte Carlo method; UI/NCI, usual intake by the National Cancer Institute method; NHANES, National Health and Nutrition Examination Survey. ^2^ Regression coefficient (β) across deciles of intake of added sugars. ^3^ *p* value for estimating the probability of rejecting the null hypothesis that the slope of the association is zero (β = 0) when it is true; *p* ≤ 0.001 deemed significant.

**Table 3:** Percentage of the population above the Adequate Intake of potassium by deciles of added sugars intake by various methods, the NHANES 2011–2014. ^1^

Decile	1 Day Intake	2 Day Average Intake	UI/NCI	UI/MCMC
Children (2–18 years)				
1	0.76 ± 0.19	0.74 ± 0.18	1.93 ± 0.60	4.96 ± 1.31
2	1.24 ± 0.38	1.27 ± 0.49	2.26 ± 0.56	1.04 ± 0.37
3	1.27 ± 0.49	1.81 ± 0.42	1.07 ± 0.43	0.44 ± 0.20
4	1.38 ± 0.47	1.13 ± 0.35	0.64 ± 0.27	0.65 ± 0.29
5	1.36 ± 0.39	1.03 ± 0.29	1.21 ± 0.43	0.33 ± 0.11
6	0.92 ± 0.27	0.62 ± 0.22	0.43 ± 0.18	0.32 ± 0.12
7	0.52 ± 0.27	0.46 ± 0.17	0.19 ± 0.12	0.39 ± 0.16
8	0.45 ± 0.23	0.53 ± 0.22	0.25 ± 0.13	0.29 ± 0.16
9	0.56 ± 0.23	0.36 ± 0.16	0.08 ± 0.05	0.11 ± 0.05
10	0.11 ± 0.06	0.09 ± 0.05	0.01 ± 0.03	0.07 ± 0.04
β ^2^	−0.16 ± 0.02	−0.15 ± 0.02	−0.13 ± 0.03	−0.07 ± 0.02
*p* ^3^	<0.0001	<0.0001	0.0008	0.0073
Adults (≥19 years)				
1	1.86 ± 0.37	2.41 ± 0.71	2.38 ± 0.50	2.69 ± 0.51
2	3.76 ± 0.70	3.46 ± 0.63	3.24 ± 0.65	2.66 ± 0.50
3	4.75 ± 0.71	5.41 ± 0.84	4.77 ± 0.81	3.11 ± 0.57
4	4.08 ± 0.57	3.83 ± 0.71	3.72 ± 0.64	3.57 ± 0.53
5	3.59 ± 0.74	3.28 ± 0.64	3.65 ± 0.67	2.51 ± 0.69
6	3.48 ± 0.57	3.32 ± 0.50	2.90 ± 0.37	3.14 ± 0.47
7	2.40 ± 0.66	2.24 ± 0.47	2.37 ± 0.52	3.10 ± 0.60
8	1.39 ± 0.24	2.00 ± 0.55	2.07 ± 0.55	3.60 ± 0.49
9	1.03 ± 0.31	1.11 ± 0.22	1.13 ± 0.22	2.97 ± 0.47
10	0.44 ± 0.11	0.39 ± 0.10	0.34 ± 0.10	1.97 ± 0.14
β ^2^	−0.33 ± 0.08	−0.47 ± 0.7	−0.42 ± 0.08	−0.31 ± 0.07
*p* ^3^	0.0045	0.0002	0.0005	0.0029

^1^ Values are presented as the mean ± SD unless otherwise indicated. UI/MCMC, usual intake by the Markov Chain Monte Carlo method; UI/NCI, usual intake by the National Cancer Institute method; NHANES, National Health and Nutrition Examination Survey. ^2^ Regression coefficient (β) across deciles of intake of added sugars. ^3^ *p* value for estimating the probability of rejecting the null hypothesis that the slope of the association is zero (β = 0) when it is true; *p* ≤ 0.001 deemed significant.

**Table 4:** Percentage of the population above the Adequate Intake of dietary fiber by deciles of added sugars intake by various methods, the NHANES 2011–2014. ^1^

Decile	1 Day Intake	2 Day Average Intake	UI/NCI	UI/MCMC
Children (2–18 years)				
1	1.26 ± 0.51	1.48 ± 0.71	2.27 ± 0.88	4.00 ± 1.03
2	2.42 ± 0.53	3.18 ± 0.74	2.25 ± 0.64	1.57 ± 0.56
3	1.52 ± 0.56	1.27 ± 0.45	1.88 ± 0.49	1.13 ± 0.39
4	1.57 ± 0.45	1.72 ± 0.51	1.06 ± 0.54	1.21 ± 0.48
5	1.11 ± 0.35	0.82 ± 0.38	1.59 ± 0.66	0.70 ± 0.33
6	0.92 ± 0.35	0.75 ± 0.28	0.67 ± 0.25	0.76 ± 0.26
7	0.90 ± 0.41	0.54 ± 0.26	0.46 ± 0.17	0.95 ± 0.36
8	0.58 ± 0.20	0.47 ± 0.22	0.39 ± 0.18	0.46 ± 0.26
9	0.14 ± 0.08	0.17 ± 0.07	0.19 ± 0.10	0.28 ± 0.15
10	0.03 ± 0.03	0.01 ± 0.04	0.03 ± 0.02	0.28 ± 0.13
β ^2^	−0.21 ± 0. 0.2	−0.21 ± 0.03	−0.20 ± 0.02	−0.16 ± 0.3
*p* ^3^	<0.0001	<0.0001	<0.0001	0.0019
Adults (≥19 years)				
1	6.74 ± 1.2	8.42 ± 1.41	7.16 ± 1.28	17.6 ± 2.0
2	12.2 ± 1.3	12.2 ± 1.6	11.0 ± 1.9	12.8 ± 1.2
3	13.2 ± 2.0	14.3 ± 2.1	13.1 ± 1.8	13.3 ± 1.4
4	14.1 ± 1.7	14.3 ± 2.0	13.5 ± 1.7	15.6 ± 2.1
5	10.9 ± 1.4	14.4 ± 1.4	13.1 ± 1.8	7.31 ± 1.03
6	11.6 ± 1.2	10.9 ± 1.6	11.3 ± 1.6	5.04 ± 0.94
7	8.11 ± 1.16	7.19 ± 1.10	6.55 ± 1.16	4.75 ± 0.82
8	5.37 ± 0.67	5.57 ± 0.90	5.47 ± 0.70	4.64 ± 0.91
9	3.36 ± 0.47	3.75 ± 0.45	3.12 ± 0.34	2.87 ± 0.69
10	0.82 ± 0.19	0.66 ± 0.13	0.50 ± 0.09	2.96 ± 0.68
β ^2^	−1.43 ± 0.26	−1.66 ± 0.26	−1.54 ± 0.27	−1.39 ± 0.21
*p* ^3^	0.0007	0.0002	0.0004	0.0002

^1^ Values are presented as the mean ± SD unless otherwise indicated. UI/MCMC, usual intake by the Markov Chain Monte Carlo method; UI/NCI, usual intake by the National Cancer Institute method; NHANES, National Health and Nutrition Examination Survey. ^2^ Regression coefficient (β) across deciles of intake of added sugars. ^3^ *p* value for estimating the probability of rejecting the null hypothesis that the slope of the association is zero (β = 0) when it is true; *p* ≤ 0.001 deemed significant.

**Table 5:** Percentage of the population below the Estimated Average Requirement of vitamin D by deciles of added sugars intake by various methods, the NHANES 2011–2014. ^1^

Decile	1 Day Intake	2 Day Average Intake	UI/NCI	UI/MCMC
Children (2–18 years)				
1	91.4 ± 1.5	89.8 ± 2.6	90.5 ± 2.1	92.0 ± 2.4
2	88.2 ± 2.3	90.0 ± 2.0	88.6 ± 2.0	85.5 ± 1.4
3	85.3 ± 1.9	89.6 ± 2.6	87.4 ± 2.4	93.0 ± 1.3
4	87.7 ± 2.5	87.8 ± 2.0	88.6 ± 1.9	93.7 ± 1.5
5	90.3 ± 1.9	89.4 ± 2.2	90.6 ± 1.5	89.6 ± 1.3
6	93.9 ± 1.5	93.4 ± 1.6	92.2 ± 1.4	92.6 ± 1.5
7	94.7 ± 1.4	94.7 ± 1.4	94.2 ± 1.5	91.8 ± 2.0
8	94.5 ± 1.3	93.6 ± 1.7	93.1 ± 1.6	90.3 ± 1.5
9	95.6 ± 0.9	96.7 ± 1.0	97.0 ± 0.9	93.5 ± 1.4
10	98.6 ± 0.4	98.7 ± 0.5	98.5 ± 0.5	94.2 ± 1.0
β ^2^	1.22 ± 0.19	1.29 ± 0.15	1.32 ± 0.14	0.55 ± 0.22
*p* ^3^	0.0002	<0.0001	<0.0001	0.0378
Adults (≥19 years)				
1	96.8 ± 0.9	96.5 ± 0.8	96.4 ± 0.7	92.9 ± 1.1
2	94.3 ± 1.1	94.6 ± 1.1	95.2 ± 0.8	93.1 ± 0.8
3	91.2 ± 1.1	93.3 ± 1.0	93.0 ± 1.2	93.7 ± 0.7
4	92.1 ± 1.3	93.4 ± 1.1	91.7 ± 1.4	94.9 ± 1.2
5	93.0 ± 1.1	93.7 ± 0.9	93.3 ± 1.0	94.4 ± 0.7
6	93.9 ± 0.8	92.9 ± 1.0	94.1 ± 0.9	94.8 ± 0.9
7	94.7 ± 0.7	93.8 ± 1.4	92.4 ± 1.2	94.9 ± 0.9
8	95.1 ± 0.8	94.9 ± 1.2	94.8 ± 0.8	95.3 ± 0.8
9	96.1 ± 0.8	96.4 ± 0.8	96.3 ± 0.7	95.3 ± 0.8
10	98.2 ± 0.5	98.5 ± 0.5	98.5 ± 0.4	97.0 ± 0.9
β ^2^	0.41 ± 0.20	0.39 ± 0.18	0.35 ± 0.19	0.37 ± 0.05
*p* ^3^	0.0758	0.0602	0.1051	0.0001

^1^ Values are presented as the mean ± SD unless otherwise indicated. UI/MCMC, usual intake by the Markov Chain Monte Carlo method; UI/NCI, usual intake by the National Cancer Institute method; NHANES, National Health and Nutrition Examination Survey. ^2^ Regression coefficient (β) across deciles of intake of added sugars. ^3^ *p* value for estimating the probability of rejecting the null hypothesis that the slope of the association is zero (β = 0) when it is true; *p* ≤ 0.001 deemed significant.

**Table 6:** Comparison of regression coefficients for the percentage below the Estimated Average Requirement/above the Adequate Intake by different methods of added sugars intake, the NHANES 2011–2014. ^1^

Regression Coefficient	1 Day Intake	2 Day Average Intake	UI/NCI	UI/MCMC
Children (2–18 years)				
Calcium	4.04 (1.45, 6.64)	4.19 (2.13, 6.24)	4.89 (3.18, 6.61)	7.85 (3.76, 11.93)
Potassium	−0.16 (−0.20, −0.12)	−0.15 (−0.19, −0.10)	−0.13 (−0.20, −0.06)	−0.07 (−0.13, −0.02)
Vitamin D	1.22 (0.69, 1.76)	1.29 (0.88, 1.70)	1.32 (0.92, 1.72)	0.55 (−0.06, 1.17)
Dietary fiber	−0.21 (−0.28, −0.15)	−0.21 (−0.28, −0.15)	−0.20 (−0.26, −0.14)	−0.16 (−0.25, −0.06)
Adults (≥19 years)				
Calcium	1.10 (−1.54, 3.74)	0.62 (−2.06, 3.30)	0.04 (−2.80, 2.87)	−3.40 (−5.31, −1.49) *
Potassium	−0.33 (−0.56, −0.10)	−0.47 (−0.67, −0.26)	−0.42 (−0.63, −0.21)	−0.31 (−0.51, −0.11)
Vitamin D	0.41 (−0.14, 0.96)	0.39 (−0.10, 0.89)	0.35 (−0.18, 0.87)	0.37 (0.24, 0.50)
Dietary fiber	−1.43 (−2.16, −0.70)	−1.66 (−2.39, −0.93)	−1.54 (−2.27, −0.80)	−1.39 (−1.97, −0.81)

^1^ Values are presented as β values (97.5% confidence intervals). β represents the regression coefficient for the percentage of the population below the EAR (for calcium and vitamin D) or above the AI (for potassium and dietary fiber). UI/MCMC, usual intake by the Markov Chain Monte Carlo method; UI/NCI, usual intake by the National Cancer Institute method; NHANES, National Health and Nutrition Examination Survey. * Indicates significantly different from other regression coefficients in the row (confidence intervals do not overlap).
